# Rewiring Lipid Metabolism by Targeting PCSK9 and HMGCR to Treat Liver Cancer

**DOI:** 10.3390/cancers15010003

**Published:** 2022-12-20

**Authors:** Malak Alannan, Véronique Trézéguet, Nivea Dias Amoêdo, Rodrigue Rossignol, Walid Mahfouf, Hamid Reza Rezvani, Franziska Dittrich-Domergue, Patrick Moreau, Sabrina Lacomme, Etienne Gontier, Christophe F. Grosset, Bassam Badran, Hussein Fayyad-Kazan, Aksam J. Merched

**Affiliations:** 1 Bordeaux Institute of Oncology (BRIC), INSERM U1312, University of Bordeaux, F-33000 Bordeaux, France; 2 CELLOMET, Functional Genomics Center (CGFB), INSERM U1211, University of Bordeaux, F-33000 Bordeaux, France; 3 Laboratoire de Biogenèse Membranaire, Centre National de la Recherche Scientifique (CNRS), UMR 5200, University of Bordeaux, F-33140 Villenave d’Ornon, France; 4 CNRS, INSERM, Bordeaux Imaging Center (BIC), University of Bordeaux, UAR 3420, US 4, F-33000 Bordeaux, France; 5 Laboratory of Cancer Biology and Molecular Immunology, Faculty of Sciences I, Lebanese University, Hadath, Beirut, Lebanon

**Keywords:** hepatocellular carcinoma, hepatoblastoma, lipotoxicity, peridroplet mitochondria

## Abstract

**Simple Summary:**

We targeted abnormalities in lipid metabolism of liver cancer cells and showed that tumoral addiction to lipids can be potentially overridden by available drugs. Anti-PCSK9 alone or in combination with statin treatment can live up to this challenge and disrupt the process of oncogenesis opening up new avenues for potential drug repositioning for the treatment of liver cancer.

**Abstract:**

Alterations in lipid handling are an important hallmark in cancer. Our aim here is to target key metabolic enzymes to reshape the oncogenic lipid metabolism triggering irreversible cell breakdown. We targeted the key metabolic player proprotein convertase subtilisin/kexin type 9 (PCSK9) using a pharmacological inhibitor (R-IMPP) alone or in combination with 3-hydroxy 3-methylglutaryl-Coenzyme A reductase (HMGCR) inhibitor, simvastatin. We assessed the effect of these treatments using 3 hepatoma cell lines, Huh6, Huh7 and HepG2 and a tumor xenograft in chicken choriorallantoic membrane (CAM) model. PCSK9 deficiency led to dose-dependent inhibition of cell proliferation in all cell lines and a decrease in cell migration. Co-treatment with simvastatin presented synergetic anti-proliferative effects. At the metabolic level, mitochondrial respiration assays as well as the assessment of glucose and glutamine consumption showed higher metabolic adaptability and surge in the absence of PCSK9. Enhanced lipid uptake and biogenesis led to excessive accumulation of intracellular lipid droplets as revealed by electron microscopy and metabolic tracing. Using xenograft experiments in CAM model, we further demonstrated the effect of anti-PCSK9 treatment in reducing tumor aggressiveness. Targeting PCSK9 alone or in combination with statins deserves to be considered as a new therapeutic option in liver cancer clinical applications.

## 1. Introduction

Deregulation of energetic metabolism, specifically enhanced lipid biosynthesis, is an emerging hallmark of many cancers including the adult and pediatric forms of liver cancer, hepatocellular carcinoma (HCC) and hepatoblastoma (HB), respectively. HCC is the most frequent primary malignant liver disease that ranks the third in cancer-related deaths worldwide [1]. HB, on the other hand, is a rare malignant embryonic tumor affecting children aged 3 years and younger [2]. Despite of the medical advances and treatments available for both tumors, their incidence has been increasing drastically over the past decade, thus triggering researchers to find alternative therapeutic approaches to better target these diseases.

The addiction of cancer cells to lipid ensures the energetic needs of the cells, building blocks for membranes as well as signaling molecules to drive and sustain oncogenesis [3]. Since defects in hepatic lipid metabolism rewire many cellular pathways involved in oncogenesis and metastasis, interfering with this metabolism within the tumor and surrounding microenvironment becomes an attractive therapeutic approach for treating liver cancer patients. Because of the flexibility in the metabolic needs of cancer cells and the complex interplay among these key players of lipid metabolism, some factors may be more valuable and more relevant therapeutic targets.

One of these key enzymes is the proprotein convertase subtilisin/kexin type 9, or PCSK9. After the discovery of its critical role in regulating lipid metabolism, many therapeutic approaches targeting PCSK9 have been used in clinic mainly in combination with statins (inhibitors of HMGCR) to lower the hypolipidemic threshold in patients suffering from hyperlipidemia and cardiovascular diseases. Among these are monoclonal antibodies and anti-PCSK9 small interfering ribonucleic acid (siRNA). Different approaches called anti-secretagogue were developed to inhibit PCSK9 translation (by stalling of human 80S ribosomal subunit) and secretion [4]. Two molecules have been discovered, (R)-*N*-(isoquinolin-1-yl)-3-(4-methoxyphenyl)-*N*-(piperidin-3-yl) propanamide (R-IMPP) and PF-06446846, which underscore the therapeutic potential behind the use of selective inhibitors of messenger RNA (mRNA) translation.

PCSK9 is now attracting more attention in oncology because its tight association with the incidence and progression of several cancers [5]. Indeed, the lipid metabolic need seems to be a common feature of many cancers. As a matter of fact, the expression of PCSK9 is deregulated in many types of cancers such as neuroglioma, breast cancer and colorectal cancer [5,6]. Interestingly, HCC tumor tissues presented high expression of PCSK9, which is correlated with poor prognosis after curative resection. This metabolic feature seemed to be an independent risk factor for overall and disease-free survivals [7].

**Figure 1:**
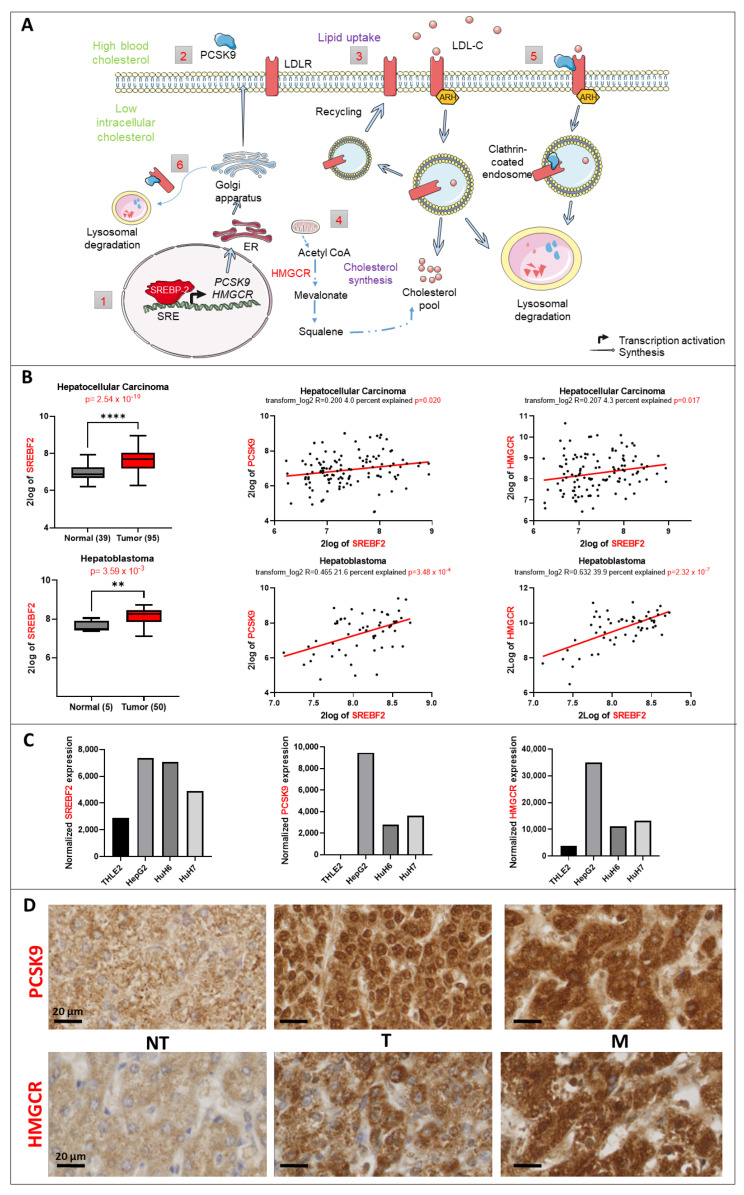
Lipid metabolic changes in liver cancer. (**A**) Control of the lipid metabolism by the transcription factor SREBP2. (1) SREBP2 is a transcription factor that regulates the expression of *PCSK9* and *HMGCR* genes (2) The synthesis of PCSK9 is regulated transcriptionally by SREBP-2. Once translated, it will travel through the endoplasmic reticulum (ER) and Golgi apparatus for maturation before being secreted. (3) Lipid uptake: In response to high blood cholesterol, LDLR expressed on the cell surface will bind to LDL-cholesterol (LDL-C) and will be internalized in an autosomal recessive hypercholesterolemia (ARH)-dependent manner via clathrin-coated endosomes. Inside the endosomes, LDL-C will dissociate from the receptor and will be directed toward lysosomal degradation, whilst LDLR will be recycled back to the cell surface. (4) Cholesterol synthesis: HMGCR is the rate-limiting enzyme that catalyzes the de novo synthesis of cholesterol to increase its levels in response to a decrease in the intracellular levels of cholesterol. (5) Extracellular and (6) intracellular pathway regulation of LDLR by PCSK9: in both pathways, PCSK9 binds to the epidermal growth factor (EGF)-like domain of LDLR and targets it to lysosomal degradation instead of recycling. (**B**) *SREBF2* gene expression upregulation in 2 transcriptomic datasets and its correlation to *PCSK9* and *HMGCR* genes generated from R2: Genomics Analysis and Visualization Platform “http://r2.amc.nl” (accessed on 23 September 2022). Upper histogram, hepatocellular carcinoma (HCC): Wu—134—MAS 5.0—u133p2. Lower histogram, hepatoblastoma (HB): López-Terrada—55—fRMA—u133p2. Unpaired *t*-test, ** *p* < 0.01; **** *p* < 0.0001. Each histogram is followed by two-tailed pearson correlation which shows a positive correlation between *SREBF2* and *PCSK9* as well as *SREBF2* and *HMGCR* gene expression in the same datasets. (**C**) Normalized gene expression of *SREBF2*, *PCSK9* and *HMGCR* in three liver cancer cell lines (HepG2, Huh6 and Huh7) and one normal cell line (THLE2). (**D**) PCSK9 and HMGCR staining of HB tumoral (T) tissues were compared to non-tumoral (NT) tissues from the same patients. The staining in metastatic (M) tissues (diaphragm) was also checked. Samples were collected as described in materials and methods. The black bars represent 20 µm.

At the functional level, PCSK9 is involved in the degradation of the hepatic low density lipoprotein receptor (LDLR) and other members of LDLRs such as very low-density lipoprotein receptor (VLDLR) and the apolipoprotein E receptor 2 (ApoER2) [8]. The promotor of *PCSK9* gene contains a functional sterol regulatory element (SRE) that is targeted by transcription factor called sterol-responsive element binding protein 2 (SREBP2) in response to any change in the intracellular levels of cholesterol (Figure 1A) [9]. SREBP2 regulates the synthesis and absorption of cholesterol as well by targeting the gene expression of *HMGCR*, HMG-CoA synthase (*HMGCS*), farnesyl diphosphate synthase (*FDPS*) and squalene synthase (*FDFT1*) [10].

Our initial observation of defective expression of many enzymes and factors involved in lipid metabolism in liver cancers such as SREBP2 transcription factor led us to focus on its main targets, PCSK9 and HMGCR. The present study aimed to assess the interest of inhibiting PCSK9 and HMGCR in liver cancer using different in vitro and in vivo experimental approaches. We showed that inhibiting PCSK9 alone or in combination with HMGCR inhibition was capable of rewiring lipid metabolism of liver cancer cells, impairing their growth, migration and energetic metabolism.

## 2. Materials and Methods

### 2.1. Transcriptomic Data Acquisition

The R2: Genomics Analysis and Visualization Platform “http://r2.amc.nl” (accessed on 23 September 2022) was used to generate the gene expression data from different available datasets. By logging in to this platform, the expression level of a gene of interest in a specific type of disease can be checked. In this study, two different datasets were selected: Hepatoblastoma—López-Terrada—55—fRMA—u133p2 (GEO ID: gse75271) [11] and Tumor HCC—Wu—134—MAS50 (GEO ID: gse45436) [12]. The expression of lipid-related genes such as *PCSK9*, *SREBF2* and *HMGCR* and the correlation between them were checked. Numeric data of gene expression were downloaded in excel files and graphs were generated using GraphPad Prism 9 software (GraphPad Software, Inc., San Diego, CA, USA). In addition, the level of expression of lipid-related genes in hepatic cell lines was generated by referring to the transcriptomic data analysis conducted on these cell lines by our team [13]. 

### 2.2. Cell Culture 

Human HCC (Huh7)- and HB (Huh6, HepG2)-derived cell lines were cultured in Dulbecco’s Modified Eagle Medium (DMEM GlutaMAX™ supplemented, with high (4.5 g/L) for Huh7 and HepG2 or low (1 g/L) D-glucose for Huh6) (Gibco, Invitrogen), supplemented with 10% fetal bovine serum (FBS), 100 µg/mL streptomycin and 100 U/mL penicillin. The cells were maintained at 37 °C in a humidified atmosphere of 5% CO_2_. Cell line authentication was performed on April 2021 using short tandem repeats (LGC, Molsheim, France) and the absence of mycoplasma contamination was tested on a monthly basis.

The Huh7 cell line originates from male hepatoma tissue that was surgically removed from a 57-year-old Japanese male in 1982 [14]. The Huh6 cell line originates from the liver hepatoblastoma of a 12-month-old Japanese male in 1985 [15]. Both cell lines were purchased from Japanese Collection of Research Bioresources (JCRB) Cell Bank. HepG2 is derived from liver hepatocellular carcinoma of a 15-year-old Caucasian male in 1975 [16]. It was purchased from the American Type Culture Collection (ATCC, Rockville, MD, USA).

### 2.3. RNA Sequencing Analysis

Total RNA from Huh6, Huh7, HepG2 and THLE-2 cell lines was extracted using the *mir*Vana kit (Thermo Fisher Scientific) according to the supplier’s protocol and the analysis was carried out by Hooks et al. [13] in a manner similar to what they carried out for patient tissues.

### 2.4. Lentivirus Production and Transduction

Lentivirus vector production was carried out by the Vect’UB service platform, (INSERM US 005-CNRS UMS 3427-TBM-Core, Université de Bordeaux, France). Lentiviral particles were produced by transient transfection of HEK293T (human embryonic kidney cells) according to standard protocols. In brief, subconfluent HEK293T cells were co-transfected with lentiviral genome (psPAX2) (gift from Didier Trono (Addgene plasmid # 12260), with an envelope coding plasmid (pMD2G-VSVG) and with vector constructs (305 pLKO-sh886 or 306 pLKO-shCTR) by calcium phosphate precipitation. LVs were harvested 48 hours’ post-transfection and concentrated by ultrafiltration, Viral titers of VSV-g pseudotype pLV lentivectors were determined by transducing HEK293T cells with serial dilutions of viral supernatant and lentiviral integration was evaluated by quantitative-PCR using RRE primers. The following forward (F) and reverse (R) sequences of shPCSK9-886 were used:

F-5′ CCGGGGGTCATGGTCACCGACTTCG*CTCGAG*CGAAGTCGGTGACCATGACCCTTTTT-3′ and R-5′ AATTCAAAAAGGGTCATGGTCACCGACTTCG*CTCGAG*CGAAGTCGGTGACCATGACCC 3′. The hairpin sequence of negative control shRNA is:

CCTAAGGTTAAGTCGCCCTCGCTCGAGCGAGGGCGACTTAACCTTAGG “http://www.addgene.org/pgvec1?f=c&identifier=1864&atqx=plko&cmd=findpl” (accessed on 15 January 2021)

Stable inhibition of PCSK9 expression was induced by cell transduction with the lentivirus 305 pLKO-sh886 (shPCSK9) or the control (306 pLKO-shCTR) at an MOI of 10. Transduced cells were selected using puromycin (P8833, Sigma-Aldrich, St. Louis, MO, USA) at 3 μg/mL.

### 2.5. Immunohistochemistry (IHC)

The 3.5-µm thick sections of hepatoblastoma tumors were de-paraffinized, rehydrated and antigen retrieval was performed in 0.01 M citrate buffer pH 6 solution. All staining procedures were performed by an autostainer (Dako-Agilent, Santa Clara, CA, USA) using standard reagents provided by the manufacturer. The sections were blocked using EnVision™ Flex peroxidase-blocking reagent (SM801, Dako-Agilent, Santa Clara, CA, USA) to block endogenous peroxidase, then washed and incubated with rabbit anti-PCSK9 (1:100, 55206-1-AP, ProteinTech Group, Inc., Rosemont, IL, USA). Incubation in horseradish peroxidase (EnVision Flex/HRP, SM802, Dako-Agilent) was used for signal amplification. 3,3′-Diamino-benzidine (DAB, Dako-Agilent, Santa Clara, CA, USA) development was used for detecting primary antibodies by producing a crisp brown end product at the site of the target antigen. The slides were counterstained with hematoxylin, dehydrated and mounted. Each immunohistochemical run contained a negative control (buffer, no primary antibody). Sections were visualized with a Hamamatsu NANOZOOMER 2.0 HT at 20× magnification in the Photonic Unit of Bordeaux Imaging Center (BIC). 

### 2.6. siRNA Transfection

Small interfering siRNAs (si1{sense: 5′ GUGCUCAACUGCCAAGGGA[dT][dT] 3′; anti-sense: 5′ UCCCUUGGCAGUUGAGCAC[dT][dT] 3′} and si2 {sense: 5′ GGGUCAUGGUCACCGACUU[dT][dT] 3′; anti-sense: 5′ AAGUCGGUGACCAUGACCC[dT][dT] 3′}) against PCSK9 (Sigma Aldrich, St. Louis, MO, USA) were diluted in 1× siMAX dilution buffer (30 mM HEPES, 100 mM KCl, 1 mM MgCl_2_, pH 7.3, Eurofins). Hepatic cancer cells were transfected independently with 20 nM si1 or 2 or control siCTR (AllStars Negative Control siRNA, Qiagen, Hilden, Germany) using lipofectamine RNAi MAX transfection reagent (Invitrogen) according to the manufacturer’s instructions of reverse transfection. For transfection, Lipofectamine RNAi MAX was diluted 1/100th in transfection medium (OptiMEM, Gibco^TM^, Thermo Fisher Scientific, Waltham, MA, USA).

### 2.7. Chemical Inhibitors

Different inhibitors that regulate lipid metabolism pathways were bought from SelleckChemicals (Houston, TX, USA), including one HMGCR inhibitor that blocks the mevalonate pathway simvastatin (S1796), and one PCSK9 inhibitor called R-IMPP (S8420). The drugs were dissolved in Dimethyl sulfoxide (DMSO), except for simvastatin, and were stored at −20 °C. All of these drugs were tested at multiple doses in the 3 cell lines. Simvastatin requires to be manually activated by dissolving 50 mg in 1 mL of warm (50 °C) ethanol and adding 0.813 mL of 1 N NaOH. It is left for 30 min to allow the conversion of simvastatin to the active acid form. Finally, pH is adjusted to 7.2 using small quantities of 1 NHCl.

### 2.8. Proliferation Assay

Cells were seeded into 96-well plates in triplicates at various densities (3000 C/well for Huh7 and HepG2; 700–2000 C/well for Huh6) and then treated with various concentrations of simvastatin (0–100 µM) and R-IMPP (0–30 µM). The proliferation of cells was assessed for 5 days using CellTiter 96^®^ AQ_ueous_ One Solution Reagent (Promega, Madison, WI, USA) and the absorbance was recorded at 490 nm using ClarioStar (BMG Labtech, Champigny sur-Marne, France).

### 2.9. Viability and Cytotoxicity Assay

Huh7 cells at 3000 cells/well in triplicate wells were cultured for 2 days with a range of R-IMPP doses (0–30 μM). The ApoTox-Glo™ Triplex assay (Promega, UK) was used to measure Huh7 cell viability and cytotoxicity following the manufacturer’s instructions. Briefly, viability and cytotoxicity are measured by fluorescent signals produced when either live-cell or dead-cell proteases cleave added substrates GF-AFC (viability) and bis-AAF-R110 (cytotoxicity). Fluorescence of the cleaved products is proportional to either viability or cytotoxicity. GF-AFC can enter cells and is therefore only cleavable by live-cell protease, which incidentally becomes inactive when cell membrane activity is lost; bis-AAF-R110 cannot enter the cell, and is cleaved only by dead-cell protease leaked from cells lacking membrane integrity. Both cleaved substrates have different excitation and emission spectra. Fluorescence was measured at 400_Ex_/505_Em_ (viability) and 485_Ex_/520_Em_ (cytotoxicity) with a CLARIOstar microplate reader (BMG LabTech, Ortenberg, Germany).

### 2.10. Western Blot

Cells were lysed in RIPA buffer (Sigma) supplemented with protease and phosphatase inhibitor cocktails (Roche Diagnostics) and centrifuged at 13,000 rpm for 15 min at 4 °C. Protein concentration was determined using the Pierce™ BCA protein assay kit (Thermo Fisher Scientific, Waltham, MA, USA). Approximately 40 μg of proteins were loaded per lane for Western blot analyses in 4–15% precast polyacrylamide gel (BioRad, Hercules, CA, USA) and blotted onto 0.2 μm nitrocellulose membrane (BioRad). The membranes were blocked in 5% BSA in TBST (20 mM Tris, 150 mM NaCl, 0.1% Tween 20), then incubated with each of the following specific primary antibodies: sheep anti-PCSK9 (1 μg/mL, AF3888, R&D systems, Minneapolis, MN, USA), mouse anti-GAPDH HRP conjugated (1:10,000, BLE649203, BioLegend, San Diego, CA, USA) overnight at 4 °C. After incubation with the appropriate secondary antibody coupled with horseradish peroxidase (rabbit anti-sheep IgG HRP, 1:3000, 402100, Calbiochem, San Diego, CA, USA), all blots were revealed with Fusion FX (Vilber Lourmat) following incubation with the ECL reagents from BioRad. Quantification was performed using the ImageJ software (National Institutes of Health, Bethesda, MA, USA).

### 2.11. Migration Assay

To carry out this process, 2 × 10^4^ Huh7 cells were seeded per well in an IncuCyte^®^ ImageLock 96-well plate in the late afternoon (confluence ~90%). In the morning, scratch wounds of 700–800 micron wide were made using the IncuCyte^®^ WoundMaker, a 96-pin woundmaking tool. The cells were washed twice with 1× PBS, and a fresh medium containing the different drugs was added into the corresponding wells. The migration assay was monitored by the IncuCyte S3 live-cell analysis system (Essen BioScience, Ltd., Royston Hertfordshire, UK) up to 24 h, where images were obtained every 2 h.

### 2.12. Seahorse XF Cell Mito Stress Test

Huh7 cells treated with 10 μM R-IMPP for 48 h were seeded (4 replicates) in XFe96 Cell Culture Microplate (Agilent technologies, #102416-100) at 80–90% confluency in DMEM GlutaMAX™ supplemented with 10% FBS. They were incubated overnight at 37 °C in 5% CO_2_ atmosphere. XFe96 Sensor cartridge was hydrated in calibration solution overnight at 37 °C in a non-CO_2_ incubator. On the day of the experiment, the medium was removed and replaced with 160 μL of Seahorse XF DMEM Medium pH 7.4 (Agilent Technologies, #103575-100) supplemented with: (i) 1 mM pyruvate, 2 mM glutamine, 5 mM glucose [+Glc]; (ii) 2 mM glutamine [−Glc]; (iii) 1 mM pyruvate, 5 mM glucose, [-Gln], (iv) 2 mM glutamine, 40 µM BPTES (glutaminase inhibitor, Sigma, SML0601) [+Glnase Inhibitor]. Cells were incubated in a CO_2_ free incubator at 37 °C for 1 h. During that time, the compound working solutions were prepared from stocks at the following concentrations: 7.5 µM oligomycin (O4876, Sigma), 2 µM rotenone (R8875, Sigma), 8 µM antimycin (A8674, Sigma) and 7.5 µM CCCP (C2759, Sigma). 20 µL of the solutions are then loaded into the sensor cartridge in their respective ports A, B and C.

To run the assay, the software was prepared with the necessary information and plate map, which also indicates the number and order of injections. This process involves starting by inserting the sensor cartridge to calibrate it in Agilent Seahorse XFe/XF Analyzer before replacing the calibration plate with cell culture plate. The oxygen consumption rate (OCR) was measured upon the injection of prepared compound solutions into cells, based on the designed protocol. 

### 2.13. Measurement of Metabolites Consumption and Production

To evaluate lactate/glutamate production and glucose/glutamine uptake, Huh7 cells were seeded in triplicate on 96-well plates at a density of 5000 cells per well. 200 μL of DMEM (Glucose 4.5 g/L, L-Glutamine 4 mM) supplemented with 10 μM R-IMPP or DMSO were added to each well. Glucose/Glutamine consumption and Lactate/Glutamate production were measured with YSI 2950 Biochemistry Analyzer (YSI Life Sciences, Yellow Springs, OH, USA) in a time course incubation. The metabolites’ concentration was compared with free cell medium.

### 2.14. Radiolabeling Experiment

For radiolabeling experiments, the counted cells of each sample were transferred to a glass tube in 6 mL of DMEM medium. To start the reaction, 200 nmol (10 µCi) of [1-^14^C] acetate (PerkinElmer Life Sciences, Waltham, MA, USA) were added to each tube and the tubes were incubated at 37 °C in 5% CO_2_. The uptake of acetate was studied for each sample at 3 different time points (1 h, 2 h and 4 h). To stop the reaction, the samples were centrifuged at 1000× *g* for 5 min and the supernatants were removed. After addition of 2 mL chloroform/methanol (2:1, *v*/*v*), the cells were incubated overnight at −20 °C. To separate the aqueous and organic phases, 1 mL of 0.9% NaCl was added, the mixtures were centrifuged at 1000× *g* for 5 min. The organic phases were transferred to a new tube. The aqueous layer was re-extracted with 2 mL chloroform/methanol (2:1, *v*/*v*). The chloroform layers were combined and washed one time with 1 mL 0.9% NaCl. The organic phases were evaporated to dryness, re-suspended in 100 µL chloroform/methanol (2:1, *v*/*v*) and stored at −20 °C. Radiolabeled products were analyzed by thin-layer chromatography using HPTLC Silica Gel 60 plates (Merck). To separate neutral lipids, a mixture of hexane/ether/formic acid (10:5:0.5, *v*/*v*/*v*) was used as solvent. They were identified by co-migration with unlabeled standards, and quantification was carried out by autoradiography using a Storm 860 molecular imager (GE Healthcare).

### 2.15. Immunofluorescence

For mitochondria fluorescent labeling, cells were transduced by the lentivirus MitoC/YFP at MOI 10 for 24 h before siRNA transfection. We created this lentivirus by using the pcDNA-MitA1.03 plasmid with a cassette containing a chimera consisting of variants of CFP (mseCFP) and YFP (cp173-mVenus) connected by the Epsilon subunit of Bacillus subtilis F_o_F_1_-ATP synthase and designed to be targeted to mitochondria and to report ATP levels by FRET [17]. The slides were observed with confocal microscope model Leica DM6000 TCS SP5 MP at 20× or 40× magnification in the Photonic Unit of Bordeaux Imaging Center (BIC).

### 2.16. Transmission Electron Microscopy

HepG2, Huh6 and Huh7 cells transduced with shCTR and shPCSK9 were seeded in Nunc™ Lab-Tek™ 8-chamber slide system (ThermoFisher) to a confluence of 80%. The cells were fixed with 2.5% (*v*/*v*) glutaraldehyde and 4% (*v*/*v*) paraformaldehyde in 0.1 M phosphate buffer (pH 7.4) during 2 h at room temperature (RT), washed in 0.1 M phosphate buffer (pH 7.4) and then post-fixed in 1% osmium tetroxide in water for 1 h. Then samples were washed in water, dehydrated through a series of graded ethanol and embedded in a mixture of pure ethanol and epoxy resin (Epon 812; Delta Microscopy, Toulouse, France) 50/50 (*v*/*v*) for 2 h and then in 100% resin overnight at RT. The polymerization of the resin was carried out over a period of 48 h at 60 °C. Samples were then sectioned using a diamond knife (Diatome, Biel-Bienne, Switzerland) on an ultramicrotome (EM UC7, Leica Microsystems, Vienna, Austria). Ultrathin sections (70 nm) were picked up on copper grids. Grids were examined with a Transmission Electron Microscope (H7650, Hitachi, Tokyo, Japan) at 80 kV.

### 2.17. In Vivo CAM Model 

CAM is a highly vascularized extra-embryonic membrane, which performs multiple functions during embryonic development, including gas exchange [18,19,20]. In this method, fertilized embryos were received at the stage of segmentation and then incubated at 37.4 °C and 70% humidity. At day three of development, the eggshell was opened on the top and the opening sealed with medical-grade Durapore tape. At day 10 of embryonic development, 1 × 10^6^ transduced Huh6 cells (shCTR or shPCSK9) were embedded in Matrigel^®^ (growth-factor reduced, Corning, New York, NY, USA) droplets (40 µL) and deposited on the CAM. Pictures of tumor growth were obtained at days 3, 5 and 7 post-implantations using a stereomicroscope (SMZ745T) and a camera (Nikon DS-Fi2), then analyzed with the NSI Element D software (Nikon, Tokyo, Japan). At day seven, all tumors were fixed using 4% paraformaldehyde (PFA) and proceeded for photo documentation. 

### 2.18. Statistical Analysis

Statistical analyses were performed using GraphPad Prism 9 software (GraphPad Software, Inc., San Diego, CA, USA). For two-group comparison, we used the *t*-test when values are ≥15, otherwise Mann–Whitney rank sum test was used. For quantitative comparisons of more than two samples, One-way ANOVA test was used followed by Bonferroni post-test. Two-way ANOVA followed by Bonferroni post-test was used for experiments containing three groups or more at different time points. For correlation graphs, two-tailed Pearson correlation test was used. The experiments were carried out, independently, at least 3 times unless otherwise stated. In this case, n = number of independent experiments. A *p*-value of <0.05 was considered to be statistically significant. For all data in figures, *: *p* < 0.05, **: *p* < 0.01, ***: *p* < 0.001, ****: *p* < 0.0001 or exact *p*-values were indicated. All tests were two-sided.

## 3. Results

### 3.1. SREBP2 and Its Regulated Genes Are Overexprressed in Liver Cancers

Using available transcriptomic databases, we discovered important changes in the expression levels of some major players of the lipid metabolism such as *SREBF2*. This gene encodes a key transcription factor (i.e., SREBP2) that regulates expression of genes involved in cholesterol biosynthesis such as *PCSK9* and *HMGCR* (Figure 1A). The expression of this transcription factor is significantly upregulated in both adult and pediatric liver cancers according to the data from Wu et al., and Lopez-Terrada et al., posted in R2: Genomics Analysis and Visualization Platform (Figure 1B). As expected, the expression of *SREBF2* is correlated with that of *PCSK9* and *HMGCR*, for which expressions increase as well in HB and HCC (not shown). We confirmed the same tendencies for *SREBF2*, *PCSK9* and *HMGCR* expression in three liver cancer cell lines (e.g., HepG2, Huh6 and Huh7) in comparison with the normal liver cell line THLE2 (Figure 1C) [13]. We verified the higher expression levels of PCSK9 and HMGCR in HB tumor samples as well as in metastatic tumors (diaphragm) by immunohistochemistry (Figure 1D). Nonetheless, we noticed very heterogeneous staining reflecting a wide range and overlapping expression levels of these enzymes among tumoral (T) and non-tumoral (NT) tissues.

These findings suggest a specific metabolic pattern at the lipid level consisting of an increase in endogenous biosynthesis of cholesterol, as reflected by the increase in the rate-limiting enzyme of the mevalonate pathway, e.g., HMGCR. At the same time, since PCSK9 is involved in the degradation of several cell surface lipoprotein receptors, higher levels of this enzyme could reflect a possible slowdown of lipoprotein uptake by liver cancer cells. For these reasons, we aimed at reversing either oncogenic pathways by targeting both enzymes, individually and in combination, as potential approaches to disrupt the whole oncogenic process.

### 3.2. Targeting of PCSK9 Alone or in Combination with Statin Treatment Inhibits Cell Growth

**Figure 2:**
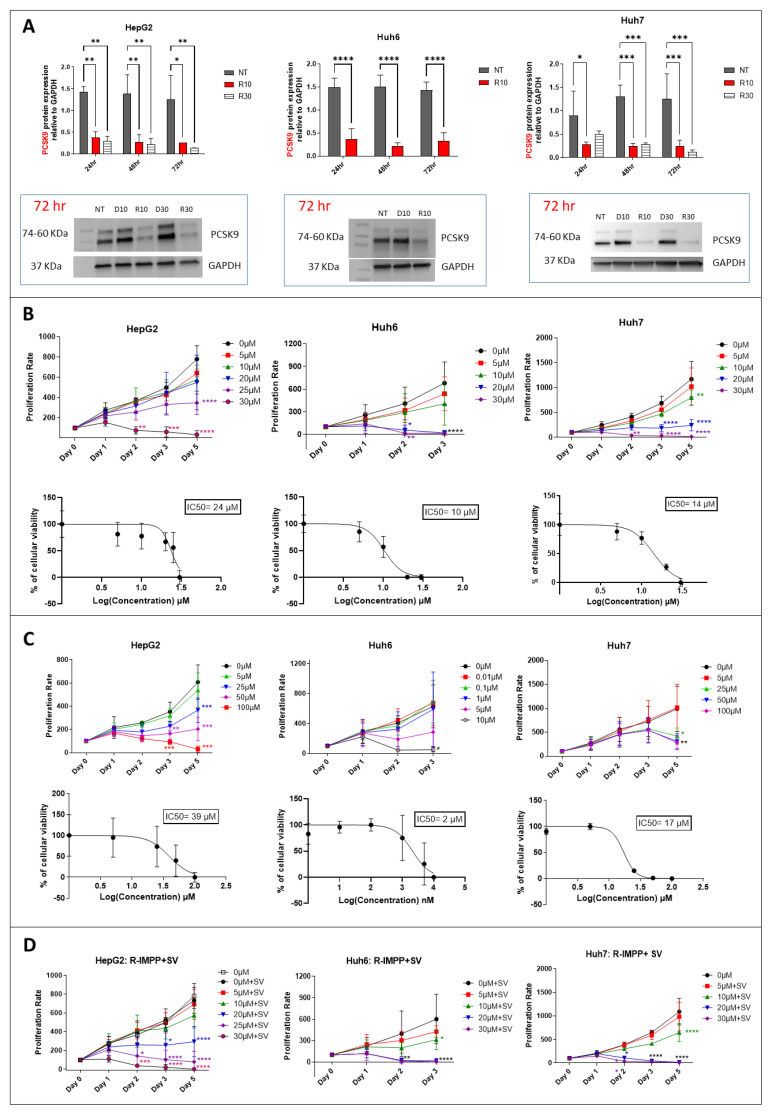
Effect of pharmacological inhibition of PCSK9 and HMGCR on cellular proliferation. (**A**) Inhibition of PCSK9 protein level by R-IMPP. Huh7 and HepG2 cells were treated with 10 and 30 μM of R-IMPP, whilst Huh6 cells were treated with 10 μM of R-IMPP. PCSK9 protein level was evaluated by Western blot in these 2 conditions 24, 48 and 72 h after treatment. Internal GAPDH was used for protein normalization (n = 3). NT: non-treated; D: DMSO; R: R-IMPP. (**B**) Dose-dependent inhibition of cell growth by R-IMPP as evaluated by MTS analyses. IC_50_ at day 3 is given for each cell line. (**C**) Proliferation was evaluated up to 5 days after SV treatment except for Huh6 where the experiment was stopped at day 3 because of the extensive cell death at this time point. IC_50_ at day 3 is given for each cell. (**D**) Effect of R-IMPP treatment in combination with SV. The concentrations of SV were selected close to the IC_50_ data from (**C**) (25 μM SV for Huh7 and HepG2 and 5 μM for Huh6). * Two-way ANOVA test. * *p* < 0.05; ** *p* < 0.01; *** *p* < 0.001; **** *p* < 0.0001. Supplementary Materials: original-images of western blot figures.

To inhibit PCSK9 pharmacologically, we used the anti-secretagogue R-IMPP [4]. First, we confirmed the strong inhibition of PCSK9 by this molecule in the Huh7 cell line, which was 81% and 92% at 10 μM and 30 μM, respectively, after 72 h of treatment (Figure 2A). Similarly, in HepG2 cell line, the inhibition of PCSK9 expression was 80 and 90% at 10 μM and 30 μM, respectively, while that in the Huh6 cell line was 77% at 10 μM, after 72 h of treatment (Figure 2A). At 30 μM of concentration, R-IMPP was very toxic for Huh6 cells (see below). Then, we assessed the effect of this drug on cell proliferation using the MTS assay (Figure 2B). Our analysis showed a dose-dependent inhibition of cell growth in all three cell lines: HepG2, Huh6 and Huh7. Huh6 was the most sensitive to the drug with an IC50 (at day 3) of 10 μM vs. 14 μM and 24 μM for Huh7 and HepG2, respectively. Interestingly, cell sensitivity to the drug seems to correlate with the expression levels of PCSK9 as seen in Figure 1C (HepG2 is the highest expressing cell line and Huh6 is the lowest one). Moreover, we obtained dose-dependent inhibition of cell proliferation by simvastatin (SV) in all cell lines (Figure 2C). Similar to the clinical approach of combining anti-PCSK9 with statins, we treated our cells with R-IMPP combined with different concentration of SV e.g., 25 μM for Huh7 and HepG2 and 5 μM for Huh6. The concentrations of SV were selected close to the IC50 data, which are 39 μM, 2 μM and 17 μM, for HepG2, Huh6 and Huh7, respectively (Figure 2C). The MTS analyses showed synergetic effects on the inhibition of cell proliferation in the presence of both drugs for the Huh7 and HepG2 cell lines but not for the Huh6 cell line, for which R-IMPP appeared to have no significant effect in the presence of SV on cell proliferation (Figure 2D). The combination drugs’ IC50 for HepG2, Huh6, and Huh7 were 17 μM, 8 μM, and 11 μM, respectively.

Therefore, we showed that inhibiting either enzymes PCSK9 or HMGCR, in which expression increases in liver cancer, was effective in reducing tumoral cell growth. We noticed some synergetic effects after combining inhibitors of both enzymes. We pursued our analysis of the effects of these treatments on other functional aspects such as cell migration and energetic metabolism.

### 3.3. Targeting PCSK9 Inhibits Cell Migration

**Figure 3:**
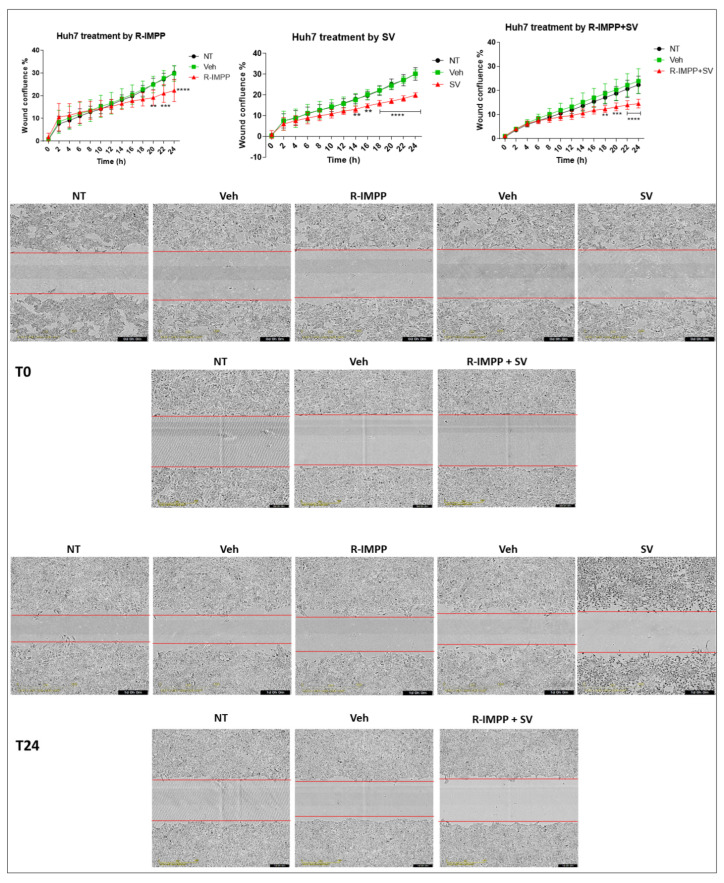
Effect of pharmacological inhibition of PCSK9 by R-IMPP on cellular migration. Effect of inhibition of PCSK9 by R-IMPP and/or HMGCR by SV on Huh7 cell migration in a wound healing assay using IncuCyte. Wound-healing confluence was followed for up to 24 h after treatment with 10 µM R-IMPP or 25 µM SV or a combination of both. NT refers to non-treated cells. Veh stands for R-IMPP (0.001% DMSO) and SV (0.001% ethanol) solvents. Two-way ANOVA test. ** *p* < 0.01; *** *p* < 0.001; **** *p* < 0.0001.

To find out more about the effects on cancer-relevant features, e.g., cell migration, we performed a wound-healing assay with the Huh7 cell line using the IncuCyte system (Figure 3). Because of the typical cell growth of HepG2 in cell clusters and Huh6 in a very sticky monolayer, the wound healing assay was not feasible in these cell lines. R-IMPP treatment at 10 μM (lower than the IC50) was effective in reducing cell migration by 26% after 24 h (Figure 3). This effect was comparable to the action of 25 μM simvastatin (33% after 24 h, Figure 3). By combining both drugs, the inhibition rate reached 36% after 24 h, indicating no significant gain compared to simvastatin alone.

Based on these results, it seems that among both targeting strategies, inhibiting PCSK9 alone was effective by itself not only in inhibiting cell proliferation but also cell migration in which simvastatin has no additive value. Taking into account these data, we decided to continue our investigations on the functional consequences of targeting PCSK9 alone.

### 3.4. Targeting PCSK9 Disrupts Cellular Bioenergetics

**Figure 4:**
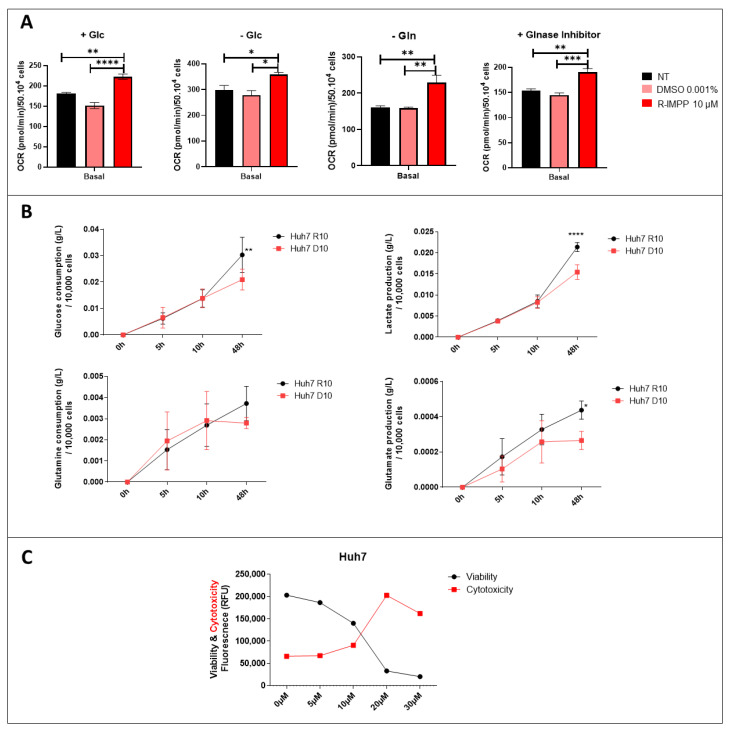
Effect of inhibition of PCSK9 on mitochondrial respiration and metabolic pathways. (**A**) OCR was evaluated by the Seahorse assay in Huh7 cells, which were incubated in different media settings, as indicated. NT: non-treated. * Ordinary one-way ANOVA test. (**B**) Glucose and glutamine consumption as well as lactate and glutamate production by Huh7 cells treated with R-IMMP (10 μM) vs. DMSO (D10) were measured at different time intervals. * Two-way ANOVA test. * *p* < 0.05; ** *p* < 0.01; *** *p* < 0.001; **** *p* < 0.0001. (**C**) Viability and cytotoxicity were measured by ApotoxGlo™ Assay in Huh7 cells treated with different doses of R-IMPP for 48 h (n = 1).

To explore energy metabolism, we measured the oxygen consumption rate (OCR) of Huh7 cell line treated with PCSK9 inhibitor using the Seahorse XF96 technology (Agilent). We performed this assay to assess mitochondrial respiration in the presence or absence of different sources of energy such as glucose and glutamine. Different experimental settings were used. The first set was conducted in the presence of complete media containing all nutrients. The second was performed in the absence of glucose, so the evaluated mitochondrial respiration is independent of glycolysis and may use a different source instead (such as glutamine). Finally, to access cell respiration dependence on glutamine, we used two conditions: one without glutamine and the second with glutaminase inhibitor. The results revealed a stimulation of OCR in anti-PCSK9-treated cells in complete medium, in the absence of glucose, of glutamine or in the presence of a glutaminase inhibitor (Figure 4A). These bioenergetics data indicate the possible stimulation of all metabolic pathways in these cells; glycolysis, oxidative phosphorylation independent of glucose and glutamine as energy substrate. These results reflect a strong metabolic flexibility of cancer cells treated with PCSK9 inhibitor to use any available resource (e.g., glucose and/or glutamine) and a higher potential to use this resource for boosting their energetic metabolism.

To further verify the metabolic use and preference of these cells, additional functional analysis was performed in these Huh7 cells treated with PCSK9 inhibitor (for 48 h) using the YSI2950 Biochemistry Analyzer. As expected, the results revealed higher glucose (to lesser extent glutamine) consumption as well as lactate and glutamate production in the absence of PCSK9 (Figure 4B), suggesting that PCSK9 deficiency strengthens the glycolytic phenotype and consumption of glutamine of tumor cells.

Cell treatment with PCSK9 inhibitor seems to play as a strong metabolic and energetic booster as translated by better adaptability to environmental metabolic resources and higher energy production. In spite of the induction of all these metabolic pathways, and the higher energy output, the treatment of liver cancer cells by anti-PCSK9 seems to be effective in reducing the viability and increasing cytotoxicity of these cells (Figure 2B and Figure 4C). Therefore, these activations seemed to lead to metabolic exhaustion and cellular toxicity. Hence, we decided to use high resolution confocal and electron microscopies to examine these metabolic manifestations at the cellular and subcellular levels.

### 3.5. PCSK9 Silencing Induces Mitochondrial ATP and Disrupts Lipid Metabolism in Cancer Cells

**Figure 5:**
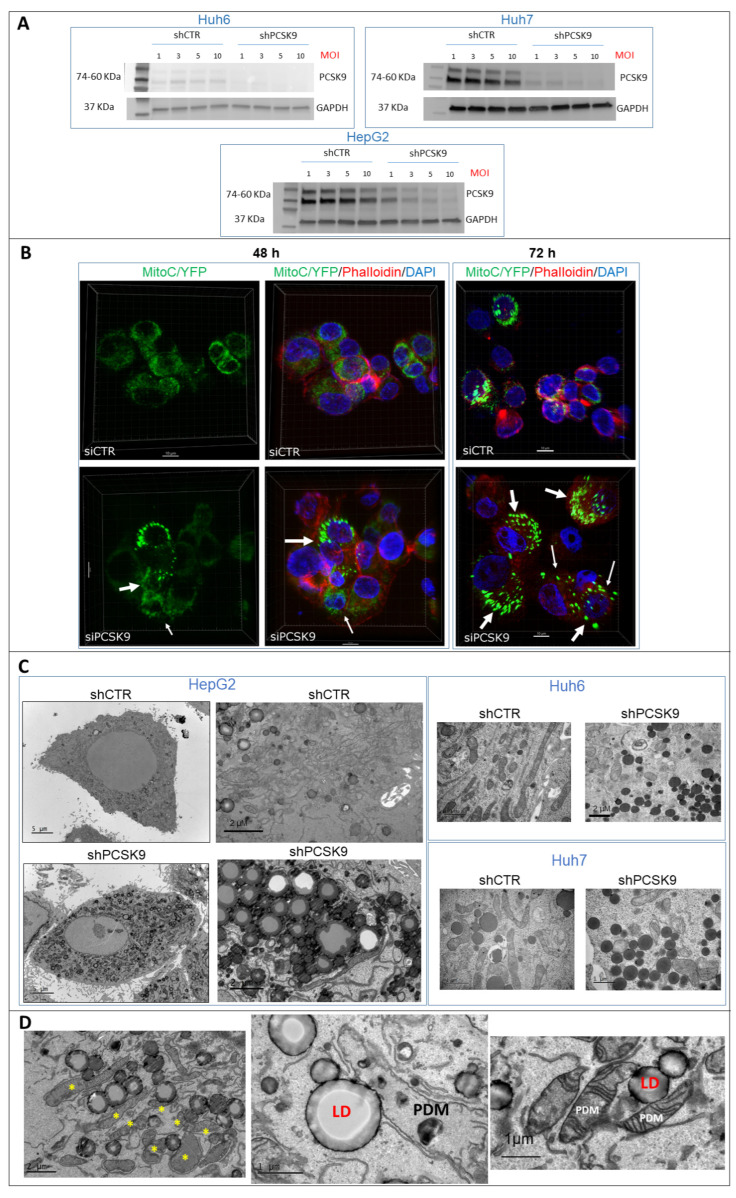
PCSK9 deficiency leads to intracellular accumulation of lipid droplets. (**A**) Validation of depletion of PCSK9 by shRNA silencing. Cell extracts were prepared after transduction with control shRNA (shCTR) or targeting PCSK9 (shPCSK9) of Huh6 or Huh7 or HepG2 cells. Different MOIs (multiplicity of infection) were tested: 1, 3, 5 and 10; the latter was picked for further experiments. A total of 40 µg of cell proteins was loaded per lane on an SDS-PAGE. After electrophoresis and transfer, the membrane was analyzed by Western blot using anti-PCSK9 antibodies and GAPDH antibodies for comparison of loading. The two bands observed for PCSK9 correspond to the pro-PCSK9 (74 kDa) and cleaved PCSK9 (60 kDa). (**B**) Confocal microscopy observation of HepG2 cells performed 48 and 72 h after transfection with PCSK9 siRNA (si1) and siCTR. Mitochondria were labeled with C/YFP using a specific lentivirus targeting system (MitoC/YFP). Phalloidin (red) and DAPI (blue) were used for microfilament and nuclear DNA staining, respectively. Arrows indicate bright mitochondria network observed more frequently when PCSK9 is silenced. (**C**,**D**) Transmission electron microscopy (TEM) photomicrographs of liver cancer cell lines transduced with specific PCSK9 shRNA (shPCSK9) or control shRNA (shCTR). Dark and dense particles seen at lower and higher magnification are lipid droplets. Stars (*) indicate PDM. Abbreviations: LD: lipid droplet; PDM: peridroplet mitochondrion. Supplementary Materials: original-images of western blot figures.

To further study the impact of PCSK9 inhibition at the morphological level, we sought a more stable approach of inhibition using shRNA or siRNA approaches. We first validated shRNA inhibition of PCSK9 production (Figure 5A). In Huh6, Huh7 and HepG2 cells, by increasing the MOI, we have a complete loss of protein expression in shPCSK9 versus shCTR transfected cells. This is best reflected at MOI 10 which was chosen for further experiments. Similarly, the siRNA approach was also validated by the same manner (not shown).

Because of the intriguing metabolic change in the mitochondrial respiration of cells in the absence of PCSK9 (Figure 4A), we sought to visually assess the level of mitochondrial adenosine triphosphate (ATP) in situ. For ATP visualization, we created a lentivirus expressing a chimeric CFP/YFP protein as FRET-based ATP probes, designed to visualize mitochondrial ATP levels in living cells [17]. ATP binding to the probe increases FRET efficiency and emission of fluorescence at 530 nm, which intensity depends on the concentration of ATP. Using confocal microscopy, we observed that some cells presented mitochondria with brighter and denser fluorescence (Figure 5B), indicating higher levels of ATP. The frequencies of these events were much higher in cells depleted for PCSK9 48 and 72 h after siRNA transfections. The fluorescence signal was more diffuse in cells transfected with the control siRNA. This observation corroborates with the increased OCR and mitochondrial respiration capacity obtained in the absence of PCSK9 (Figure 4A), which is translated by higher mitochondrial ATP production.

In addition, because the involvement of PCSK9 in maintaining adequate cellular lipid fluxes by modulating the levels of several lipid receptors, we examined PCSK9-silenced cells under a high-resolution electron microscopy to take a closer look at the cellular constituents. Thereby, the presence of lipid droplets was very obvious at lower and higher magnification by tomography electron microscopy in the absence of PCSK9 (Figure 5C). Interestingly, many of these lipid droplets were in direct interaction with mitochondria (Figure 5D), which were described as a specific type called peridroplet mitochondria (PDM) [21]. PDM are morphologically and functionally a distinct population compared to cytoplasmic mitochondria and perform different metabolic activities such as biogenesis of lipid droplets. Of note, some of these PDM seem to have smaller size and reduced cristae.

Using high resolution microscopy, we were able to confirm the surge in energetic metabolic activity as illustrated by higher mitochondrial respiration and ATP production in PCSK9-deficient liver cancer cells. Although we were expecting more lipid and lipoprotein internalization in the absence of PCSK9, we were surprised by the amount of lipid droplets accumulating in these cells. This could be the manifestation of abnormal metabolic activity and fluxes (e.g., lipid uptake with or without lipogenesis) causing higher cell toxicity linked to excessive lipid accumulation.

**Figure 6:**
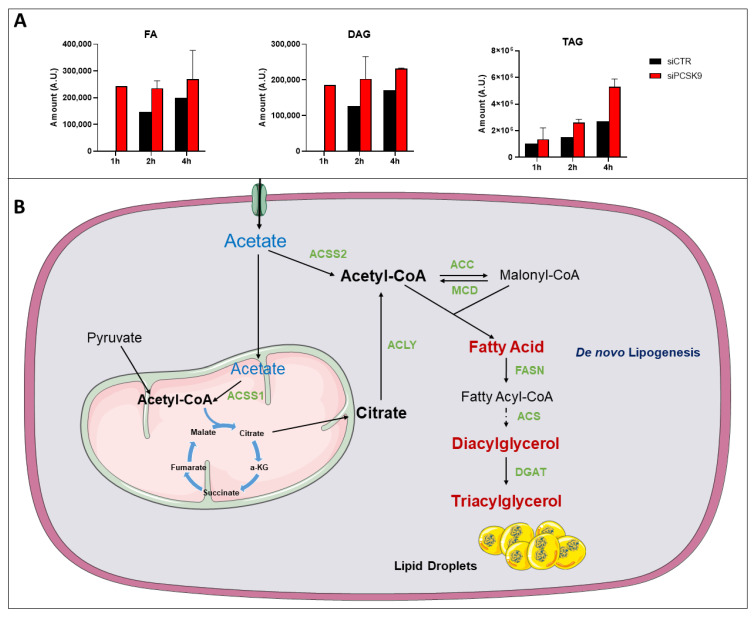
^14^C-tracing of different neutral lipids after cell feeding with [1-^14^C] acetate. (**A**) Cells were transfected with siRNA (si1 and si2) for 72 h then fed with [1-^14^C] acetate. Lipid extraction was performed 1, 2 and 4 h after [1-^14^C] acetate feeding and radiolabeled FA, DAG and TAG amounts were measured at each time point. Average values were obtained from both PCSK9 siRNA. Assays in each cell condition were performed in triplicate. (**B**) Pathway of acetate and its metabolic use to buildup lipids such as FAs up to TAG and lipid droplets. Enzymes catalyzing these reactions are presented in green. Abbreviation: ACSS1/2: Acyl-CoA synthetase short chain family member 1/2; ACC: Acetyl-CoA carboxylase; ACLY: ATP citrate lyase; MCD: Malonyl-CoA decarboxylase; FASN: fatty-acid synthase; ACS: Acetyl-CoA synthetase; DGAT: diacylglycerol acyltransferase; α-KG: α-ketoglutarate.

To investigate possible changes in lipogenesis, we added [1-^14^C] acetate to the culture media of HepG2 cells to measure the enrichment in different lipid metabolites (Figure 6). Neutral lipids including fatty acid (FA), diacylglycerol (DAG) and triacylglycerol (TAG) were extracted and quantified 1, 2 and 4 h after incubation. Lipidomic analysis showed very quick enrichment of FA and DAG starting one hour after acetate feeding in PCSK9-deficient cells (Figure 6A). The levels of these metabolites stayed relatively constant up to 4 h, while the enrichment of TAG (see metabolic pathway in Figure 6B) increased over time and reached a maximum at the 4-h time point. In control cells, acetate conversion into FA and DAG was delayed and the buildup of TAG was slower and did not reach half of its levels in cells lacking PCSK9.

Overall, these results showed a more active and elevated lipogenesis in the absence of PCSK9, suggesting that TAG accumulating in lipid droplets are originated not only from an increased uptake but also from endogenous lipid synthesis (Figure 6B).

After all these cellular experiments and data, we wanted to examine the effect of PCSK9 targeting in vivo.

### 3.6. Blocking PCSK9 Shows Anti-Tumoral Effects in CAM In Vivo Model

**Figure 7:**
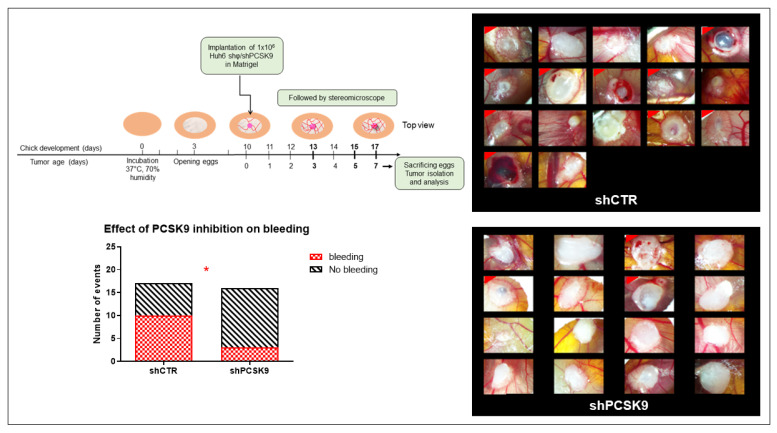
Depleting PCSK9 impairs liver tumor development in vivo. CAM model: Huh6 cells were grafted on the chicken CAM. Tumor growth was monitored on fixed tissues at day 7 after taking the photographs of tumors. The number of CAMs with tumor presenting (labeled with red corner) or not bleeding in CTR versus shPCSK9 is shown in histograms. Two-sided Fisher’s exact test, *: *p* < 0.05.

To validate the effect of blocking PCSK9 in vivo, we used the chick embryo chorioallantoic membrane (CAM) model. We have already validated this model for liver cancer with the Huh6 cell line [19]. We therefore used this specific cell line for the evaluation of the antitumor activity of shPCSK9. The Huh6 cells transduced with shPCSK9 or the empty lentivirus were deposited on the CAM and tumor growth was monitored for 7 days’ post implantation (Figure 7). Although, tumor size was not affected for the short duration of the experiment, tumors were noticeably impeded in the absence of PCSK9 compared to control tumors as assessed by the disappearance of bleeding and coagulation areas and of large vessels in tumoral tissue. The frequency of bleeding was three times higher in control tumors than in tumors lacking PCSK9 (60% vs. 20%, *p* < 0.05). The occurrence of these events reflects an aggressive tumoral phenotype [22]. Therefore, anti-PCSK9 treatment of liver cancer cells leads to a reduction in their aggressiveness during tumor development.

## 4. Discussion

Understanding the diversity of lipid metabolic networks and its cancer-associated patterns make the lipidome an attractive malleable target to be reshaped to disrupt the oncogenic process. Several available drugs targeting the lipid metabolism are showing promising anticancer properties and may emerge as new options for oncology indications [23,24].

Based on the analysis of the lipid metabolism signature of hepatic cancer cells, we targeted a specific metabolic network boosted by increased activity of PCSK9 and HMGCR enzymes. The existence of higher activity of lipogenic enzymes such as HMGCR (and other enzymes e.g., Acetyl-CoA carboxylase (ACC), fatty acid synthase (FASN), etc.) [24] and higher secretion of PCSK9, which is known to be involved in the degradation of lipoprotein receptors and keeping excessive lipid uptake at bay may, reflect a specific feature of tumor cells to rely preferably on the endogenous synthesis of lipids instead of the uptake from the outside [24].

The inhibition of both enzymes is considered as an effective approach to treat hyperlipidemia and prevent death from cardiovascular diseases. Our study aimed at assessing the potential of drug repositioning of these therapeutic approaches for the treatment of liver cancer.

Here, we showed that inhibiting either enzymes, PCSK9 or HMGCR, was effective in reducing tumoral cell growth. We noticed some synergetic effects after combining inhibitors of both enzymes. At the functional level, it seems that among the two targeting strategies, inhibiting PCSK9 alone was effective by itself not only in inhibiting cell proliferation but also cell migration in which simvastatin has no additive value.

At the metabolic level, PCSK9 deficiency was capable of inducing oxidative phosphorylation, glycolysis and consumption of glutamine of tumor cells depending on the available nutritional resources. PCSK9 inhibitor seems to play as an energetic and metabolic booster up to the toxic threshold level. Using high resolution microscopy, we were able to confirm higher mitochondrial ATP as well as excessive amount of lipid droplets accumulating in PCSK9-deficient liver cancer cells.

It was not surprising to link this “tsunami” of lipids to a drop in cell proliferation as well as increased cytotoxicity. Although we did not expect higher ATP production in these cells, lipid droplet-associated mitochondria could provide high ATP synthesis to support acyl-CoA synthesis and fatty acid esterification into triacylglycerides needed for lipid droplet expansion [21].

To brace this hypothesis, [1-^14^C] acetate feeding experiments show early and much faster conversion into fatty acids and diacylglycerol, and higher buildup into TAG in cells lacking PCSK9 (Figure 6). Acetate processing in this synthetic pathway requires the action of the Acyl-CoA synthetase short chain family member 2 (ACSS2), which produces Acetyl-CoA from acetate in a reaction that requires ATP. Therefore, the higher ATP synthesis capacity we showed here could be very well reconciled with high energy demands for Acyl-CoA synthesis in the TAG synthetic pathway and lipid droplet expansion.

As for the acetate processing enzyme ACSS2, the metabolic tracing experiment seemed to indicate higher activity in PCSK9-deficient cells. Interestingly, ACSS2 expression was reported to be negatively correlated with HCC malignancy as well as with the invasion, migration ability of HCC cells and their epithelial–mesenchymal transition (EMT) [25]. This feature may further support the anti-tumoral effect of targeting PCSK9 in liver cancer cells.

Since cancer cell addiction to lipids is widely known [26], our aim was to use this vulnerability and expose them to toxic and harmful overdose of lipids. Inhibiting PCSK9, for instance, extricates many cell surface lipoprotein receptors from degradation and opening the doors to an avalanche of lipid waves. The higher metabolic activity and excessive lipid accumulation are probably causing metabolic stress and lipotoxicity, which is widely known to induce to organellar (ER, mitochondria, lysosome) dysfunction, abnormal activation of intracellular signaling pathways, ultimately leading to cell death. Such lipid toxicity is commonly encountered in non-alcoholic fatty liver disease or NAFLD [27]. To corroborate with this eventuality, our unpublished results showed PCSK9 involvement in maintaining the redox homeostasis via the anti-oxidative KEAP1/NRF2 axis and that neutralization of PCSK9 can trigger cell death by ferroptosis.

In addition to these experimental data, PCSK9 targeting in vivo revealed a previously unknown function of PCSK9 in promoting tumor vascularization. Tumors lacking PCSK9 showed much lower bleeding events, further indicating a reduction in the aggressiveness of the tumors.

The finding that the approach of inhibiting PCSK9 alone could outperform the combinatory treatment may have interesting translational ramifications. Indeed, anti-PCSK9 therapy could be more attractive taking into account the widely known side effects and toxicity of statins [23].

## 5. Conclusions

In summary, our work here showed that blocking one branch of endogenous lipid synthesis, e.g., the mevalonate pathway by statin or unleashing lipoprotein uptake pathways by neutralizing PCSK9, were effective anti-tumoral measures. Interestingly, unlike the absence of clinical use of anti-PCSK9 alone, this approach here was sufficient to reverse many oncogenic functions of liver cancer cells, e.g., cell proliferation, migration and aggressiveness. It was capable of spurring energetic metabolism, lipid uptake and biogenesis to the extent of excessive and cytotoxic accumulation of lipids. Targeting PCSK9 alone or in combination with statins deserves to be considered as a new treatment modality and assessed in cancer clinical applications.

## Data Availability

To check the expression level of our gene of interest, two different datasets were selected: Hepatoblastoma—López-Terrada—55—fRMA—u133p2 (GEO ID: gse75271) [11] and Tumor HCC—Wu—134—MAS50 (GEO ID: gse45436) [12]. Expression levels of lipid-related genes in hepatic cell lines were generated from the transcriptomic data collection carried out by our team [13].

## Supplementary Materials

The following supporting information can be downloaded at: https://www.mdpi.com/article/10.3390/cancers15010003/s1, Supplementary Materials: original-images of western blot figures.
