# Supplementary material for: Rewiring Lipid Metabolism by Targeting PCSK9 and HMGCR to Treat Liver Cancer
Source: Cancers (Basel). 2022 Dec 20;15(1):3. doi: 10.3390/cancers15010003 (PMC9817797; doi:10.3390/cancers15010003)
Supplement: Supplementary file 1 [file cancers-15-00003-s001.zip › cancers-2022798-supplementary.pdf]

Opti-Protein Ultra Marker

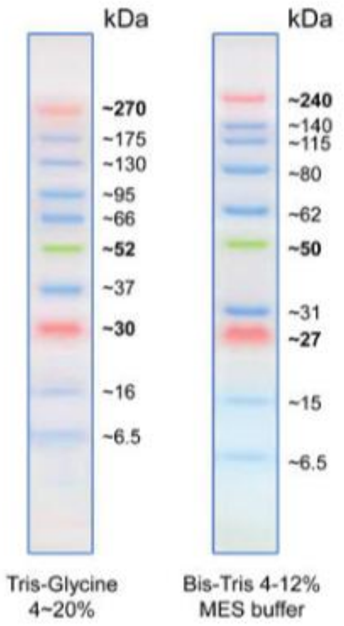

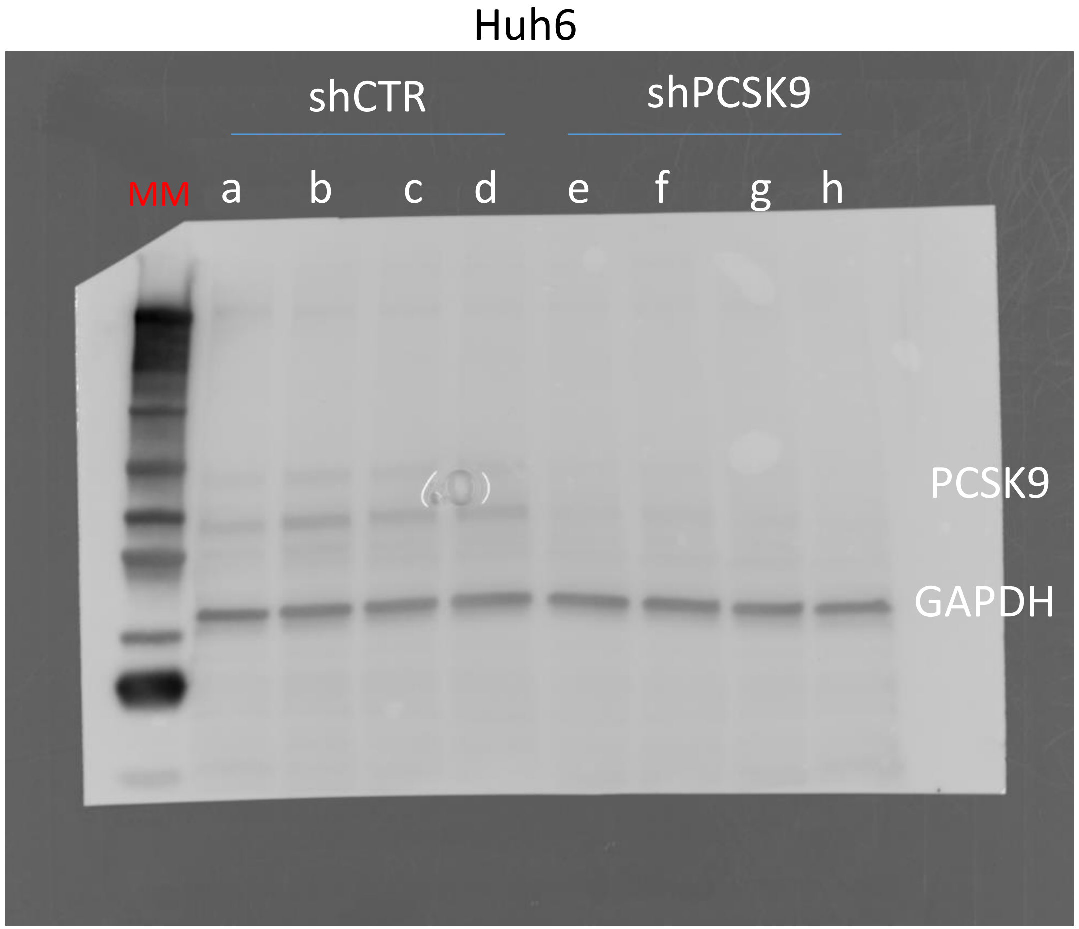

MM: molecular marker
a: Huh6 shCTR MOI 1
b: Huh6 shCTR MOI 3
c: Huh6 shCTR MOI 5
d: Huh6 shCTR MOI 10
e: Huh6 shPCSK9 MOI 1
f: Huh6 shPCSK9 MOI 3
g: Huh6 shPCSK9 MOI 5
h: Huh6 shPCSK9 MOI 10

Huh7

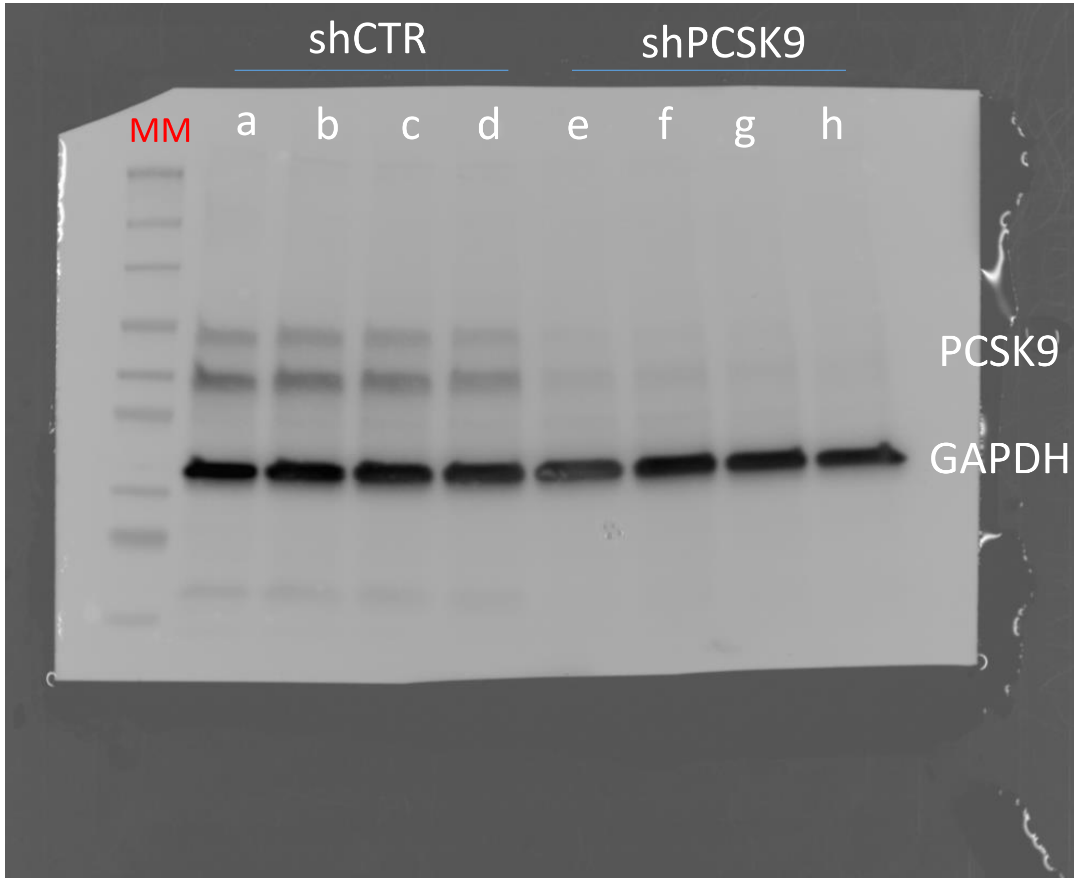

MM: molecular marker
a: Huh7 shCTR MOI 1
b: Huh7 shCTR MOI 3
c: Huh7 shCTR MOI 5
d: Huh7 shCTR MOI 10
e: Huh7 shPCSK9 MOI 1
f: Huh7 shPCSK9 MOI 3
g: Huh7 shPCSK9 MOI 5
h: Huh7 shPCSK9 MOI 10

HepG2

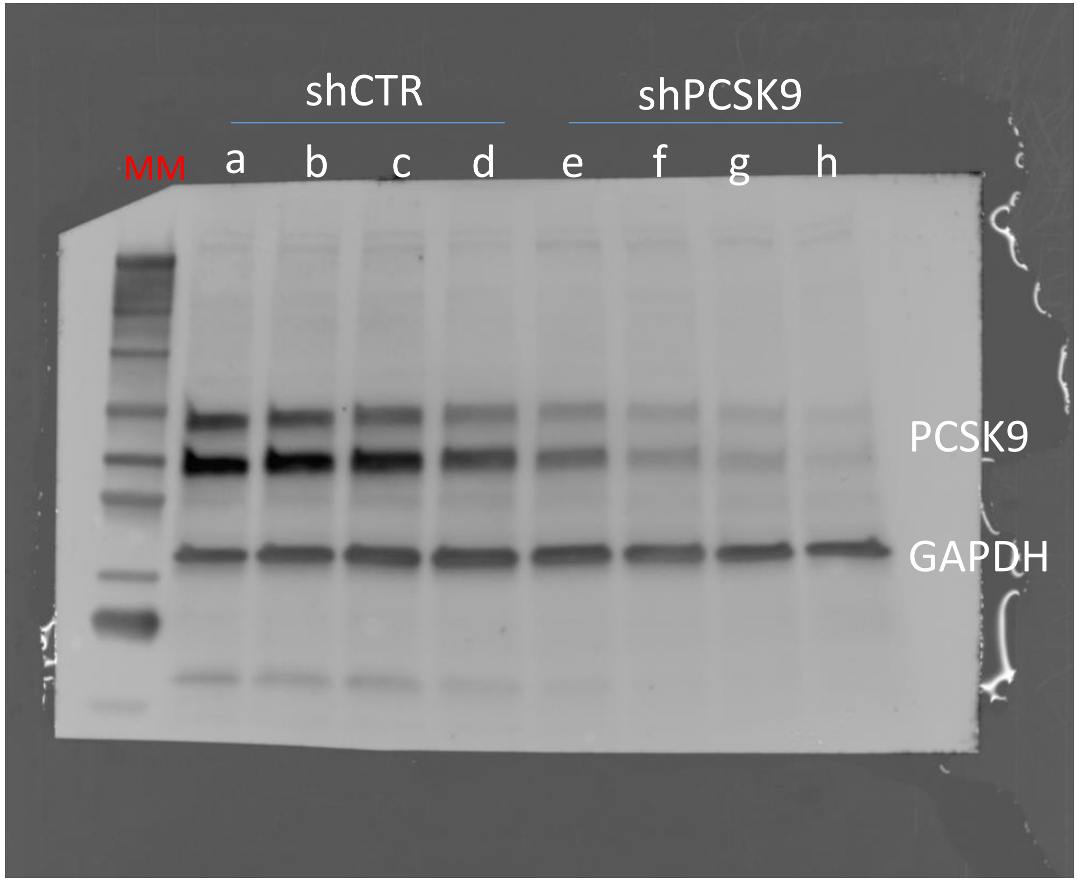

MM: molecular marker
a: HepG2 shCTR MOI 1
b: HepG2 shCTR MOI 3
c: HepG2 shCTR MOI 5
d: HepG2 shCTR MOI 10
e: HepG2 shPCSK9 MOI 1
f: HepG2 shPCSK9 MOI 3
g: HepG2 shPCSK9 MOI 5
h: HepG2 shPCSK9 MOI 10

Huh6

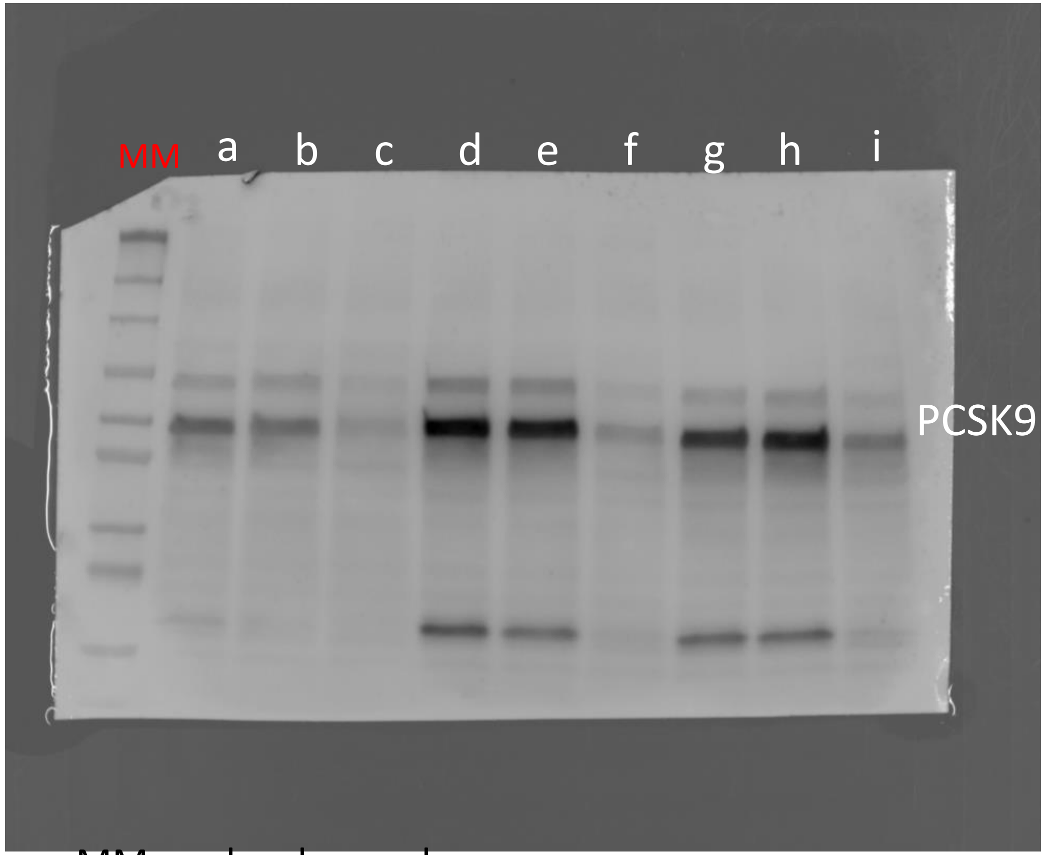

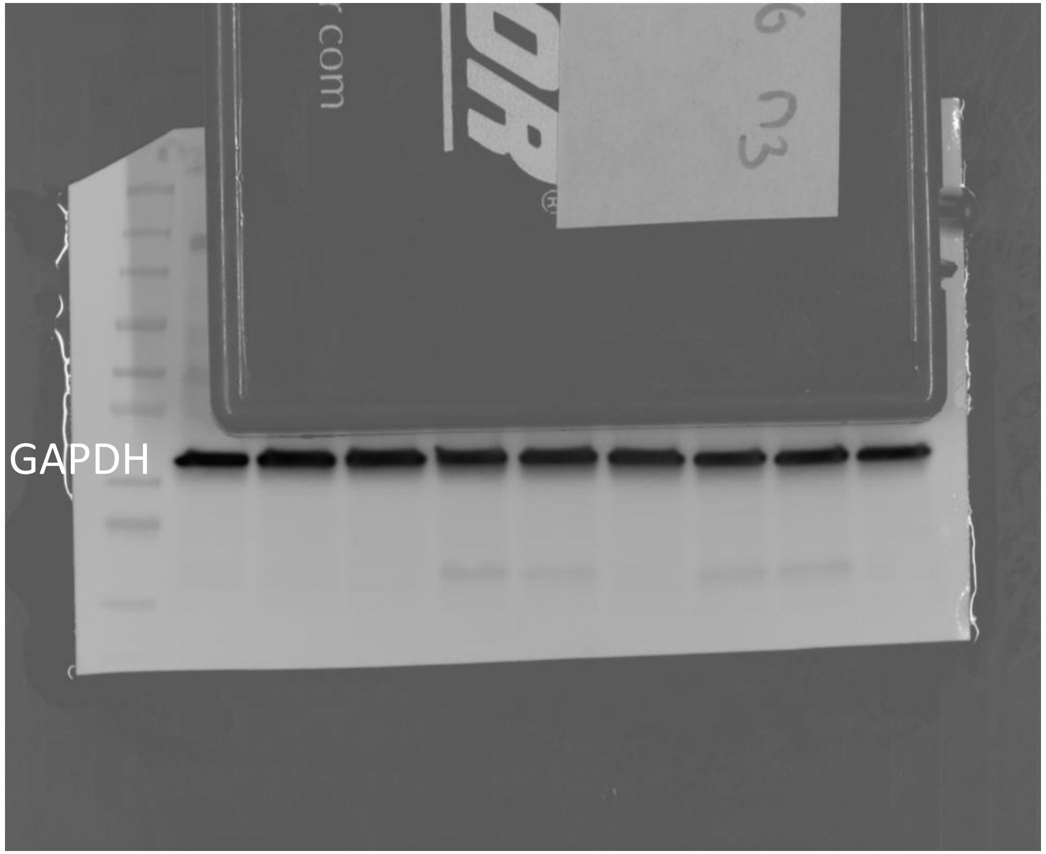

MM: molecular marker
a: Huh6 Non-treated (NT) 24hr
b: Huh6 DMSO 10 (D10) 24hr
c: Huh6 R-IMPP 10 (R10) 24hr
d: Huh6 Non-treated (NT) 48hr
e: Huh6 DMSO 10 (D10) 48hr
f: Huh6 R-IMPP 10 (R10) 48hr
g: Huh6 Non-treated (NT) 72hr
h: Huh6 DMSO 10 (D10) 72hr
i: Huh6 R-IMPP 10 (R10) 72hr

Huh7

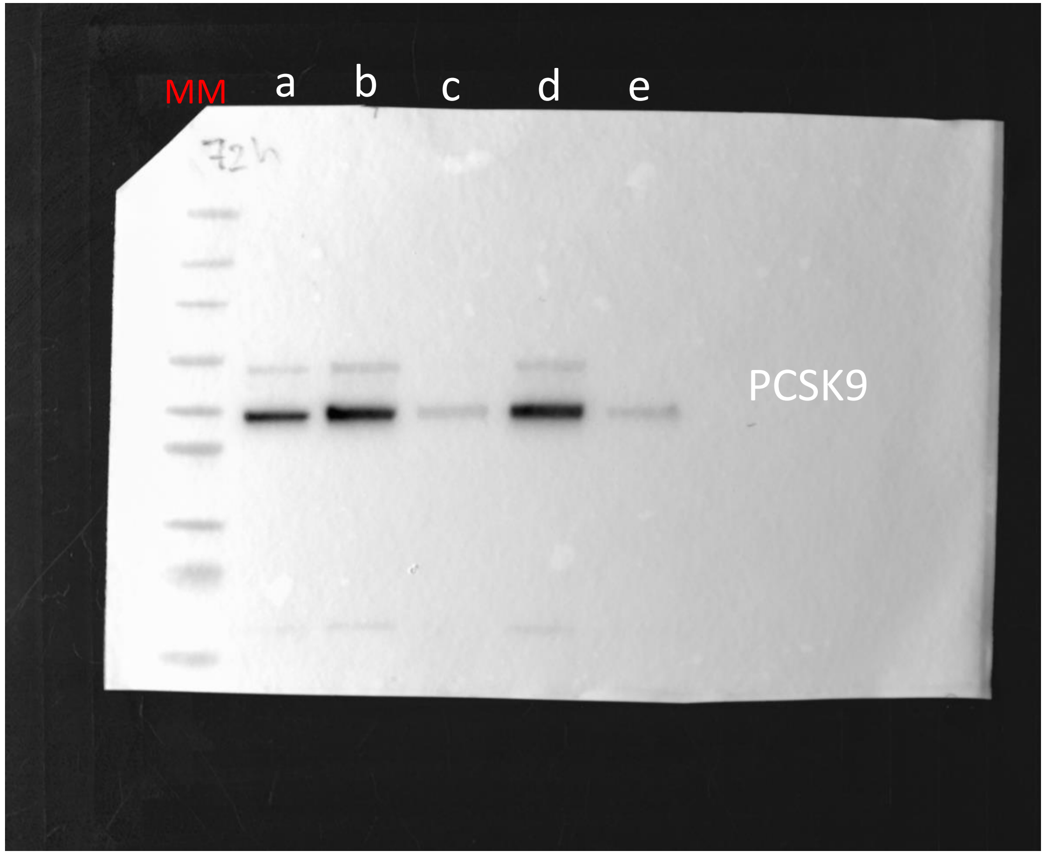

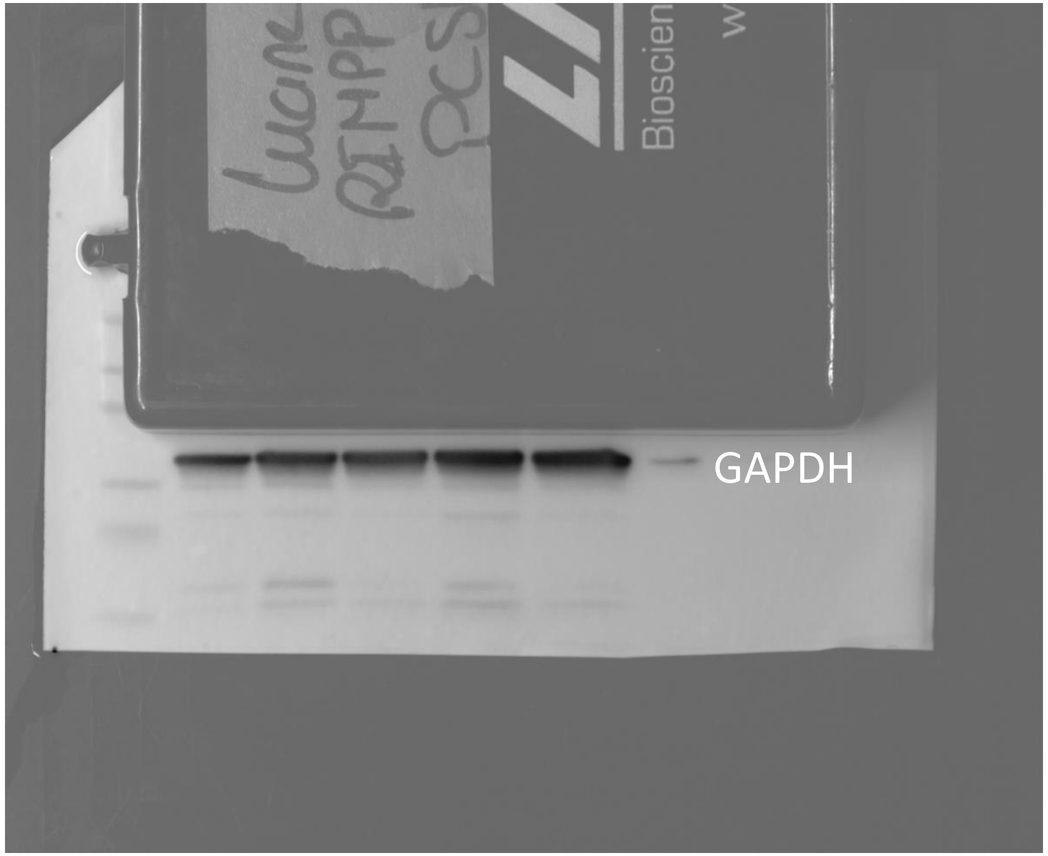

MM: molecular marker
a: Huh7 Non-treated (NT) 72hr
b: Huh7 DMSO 10 (D10) 72hr
c: Huh7 R-IMPP 10 (R10) 72hr
d: Huh7 DMSO 30 (D30) 72hr
e: Huh7 R-IMPP 30 (R30) 72hr

HepG2

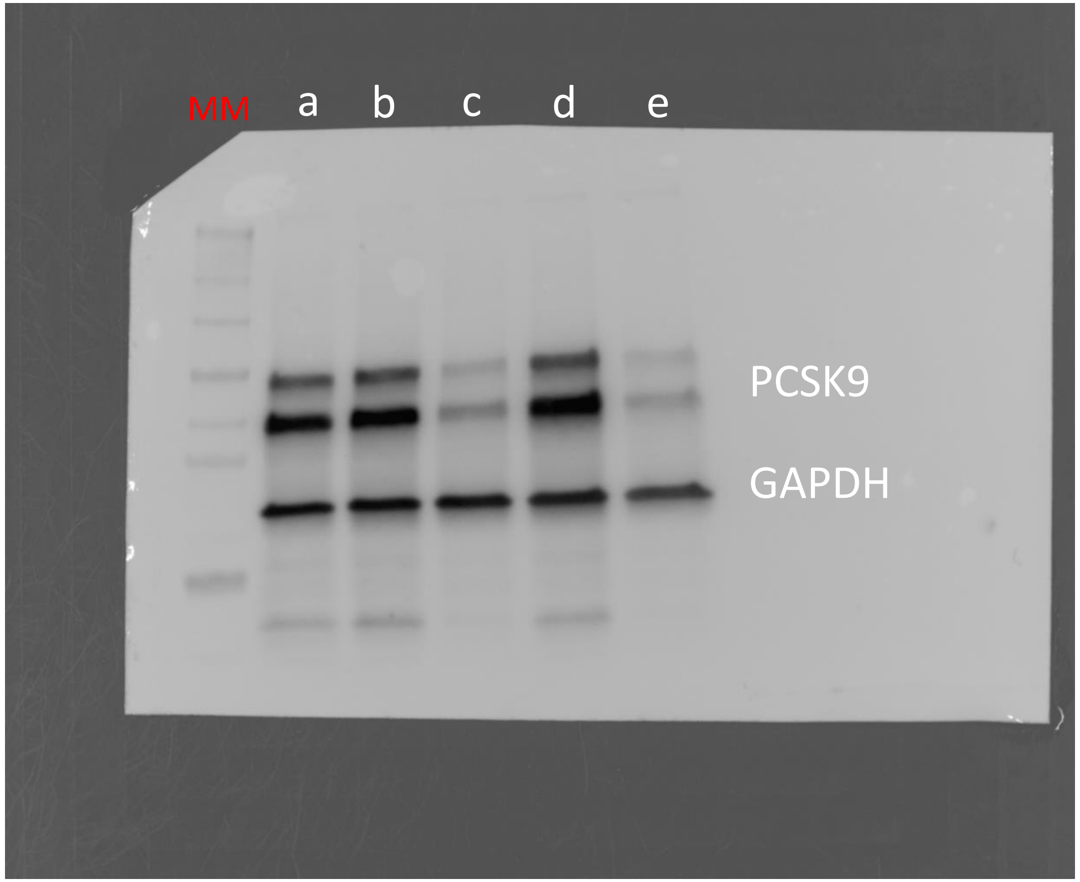

MM: molecular marker
a: HepG2 Non-treated (NT) 72hr
b: HepG2 DMSO 10 (D10) 72hr
c: HepG2 R-IMPP 10 (R10) 72hr
d: HepG2 DMSO 30 (D30) 72hr
e: HepG2 R-IMPP 30 (R30) 72hr
